# Effective p-value computations using Finite Markov Chain Imbedding (FMCI): application to local score and to pattern statistics

**DOI:** 10.1186/1748-7188-1-5

**Published:** 2006-04-07

**Authors:** Grégory Nuel

**Affiliations:** 1 Laboratoire Statistique et Génome, UEVE, CNRS (8071), INRA (1152), Evry, France

## Abstract

The technique of Finite Markov Chain Imbedding (FMCI) is a classical approach to complex combinatorial problems related to sequences. In order to get efficient algorithms, it is known that such approaches need to be first rewritten using recursive relations. We propose here to give here a general recursive algorithms allowing to compute in a numerically stable manner exact Cumulative Distribution Function (CDF) or complementary CDF (CCDF). These algorithms are then applied in two particular cases: the local score of one sequence and pattern statistics. In both cases, asymptotic developments are derived. For the local score, our new approach allows for the very first time to compute exact p-values for a practical study (finding hydrophobic segments in a protein database) where only approximations were available before. In this study, the asymptotic approximations appear to be completely unreliable for 99.5% of the considered sequences. Concerning the pattern statistics, the new FMCI algorithms dramatically outperform the previous ones as they are more reliable, easier to implement, faster and with lower memory requirements.

## 1 Introduction

The use of Markov chains is a classical approach to deal with complex combinatorial computations related to sequences. In the particular case of pattern count on random sequences, [5] named this method Finite Markov Chain Imbedding (FMCI, see [11] or [7] for a review). Using this technique it is possible to compute exact distributions otherwise delicate to obtain with classical combinatorial methods. More recently, [12] proposed a similar approach to consider local score on i.i.d. or Markovian ([13]) random sequences. Although these methods are very elegant, they could require a lot of time and memory if they are implemented with a naive approach. The authors of [6] first stated that recursive relation could be established for any particular case in order to provide an efficient way to perform the computations. We propose here to explore in detail this idea with the aim to provide fast algorithms able to compute with high numerical accuracy both CDF (cumulative distribution function) and CCDF (complementary CDF) of any general problem which can be written as a FMCI. We apply then these results to the particular cases of local score and pattern statistics. In each case, asymptotic developments are derived and numerical results are presented.

## 2 Methods

In this part, we first introduce in section 2.1 the FMCI and see the limits of naive approaches to their corresponding numerical computations. The main results are given in section 2.3 where we propose two effective algorithms able to to compute general FMCI p-values (algorithm 1) or complementary p-value (algorithm 2). The theoretical background for these algorithms is given in the section 2.2.

### 2.1 Finite Markov Chain Imbedding

Let us consider *X *= *X*_1_,...,*X*_*n *_a sequence of Bernoulli or Markov observations and *E*_*n *_an event depending on the sequence *X*. We suppose that it is possible to build from *X *an order one Markov chain *Z *= *Z*_1_,...,*Z*_*n *_on the finite state space  of size *L*. This space contains (in the order): *k *starting states denoted *s*_1_,...,*s*_*k*_, some intermediate states, and one final absorbing state *f*. The Markov chain is designed such as

ℙ(*E*_*n*_|*Z*_1 _= *s*_*i*_) = ℙ(*Z*_*n *_= *f*|*Z*_1 _= *s*_*i*_) = ∏^*n*-1^(*s*_*i*_, *f*)     (1)

where



is the transition matrix of *Z*.

If *μ *is the starting distribution of *Z*_1_, we hence get



Using this approach (and a binary decomposition of *n *- 1), it is possible to compute the p-value with *O*(log_2_(*n*) × *L*^2^) memory complexity and *O*(log_2_(*n*) × *L*^3^) time complexity. As *L *usually grows very fast when we consider more complex events *E*_*n*_, these complexities are a huge drawback of the method. Moreover, numerical precision considerations prevent this approach to give accurate results when using the relation ℙ() = 1 - ℙ(*E*_*n*_) to compute the p-value of the complementary event (as the absolute error is then equal to the relative precision of the computations).

### 2.2 Effective computations

**Proposition 1**. For all *n *≥ 1 we have



*Proof*. This trivial to establish by recurrence using matrix block multiplications.     □

We hence get the

**Corollary 2 **(direct p-value). For all *n *≥ 1 we have

for all 1 ≤ *i *≤ *k *    ℙ(*E*_*n*_|*X*_1 _= *s*_*i*_) =      and          (5)

with *y*^*n*-2 ^computable through the following recurrence relations:

*x*^0 ^= *y*^0 ^= *v *    and, for all *j *≥ 0     *x*^*j*+1 ^= *Rx*^*j *^    and     *y*^*j*+1 ^= *y*^*j*^*+x*^*j *^    (6)

*Proof*. Simply use proposition 1 to rewrite equations (1) and (3). Recurrence relations are then obvious to establish.     □

And we also get the

**Corollary 3 **(complementary p-value). For all *n *≥ 1 we have

for all 1 ≤ *i *≤ *k *    ℙ(|*X*_1 _= *s*_*i*_) =      and          (7)

with *x*^0 ^is a size *L *- 1 column vector filled with ones and with *x*^*n*-1 ^= *R*^*n*-1^*x*^0 ^which is computable through the following recurrence relation:

for all *j *≥ 0     *x*^*j*+1 ^= *Rx*^*j *^    (8)

*Proof*. ∏ being a stochastic matrix, ∏^*n*-1 ^is also stochastic, it is therefore clear that the sum of *R*^*n*-1 ^over the columns gives 1 - *y*^*n*-2 ^and the corollary is proved.     □

Using these two corollaries, it is therefore possible to accurately compute the p-value of the event or of its complementary with a complexity *O*(*L *+ *ζ*) in memory and *O*(*n *× *ζ*) in time where *ζ *is the number of non zero terms in the matrix *R*. In the worst case, *ζ *= (*L *- 1)^2 ^but the technique of FMCI usually leads to a very sparse structure for *R*. One should note that these dramatic improvements from the naive approach could even get better by considering the structure of *R *itself, but this have to be done specifically for each considered problem. We will give detailed examples of this in both our application parts but, for the moment, we focus on the general case for which we give algorithms.

### 2.3 Algorithms

Using with the corollary 2 we get a simple algorithm to compute *p *= ℙ(*E*_*n*_)

algorithm 1: direct p-value

*x *is a real column vector of size *L *- 1 and *y *a real column vector of size *k*

**initialization ***x *= (*v*_1_,...,*v*_*L*-1_)' and *y *= (*v*_1_,...,*v*_*k*_)'

**main loop **for *i *= 1...*n *- 2 do

    • *x *= *R *× *x *(sparse product)

    • *y *= *y *+ (*x*_1_,...,*x*_*k*_)'

**end **return 

and using the corollary 3 we get an even simpler algorithm to compute the *q *= 1 - *p *= ℙ()

algorithm 2: complementary p-value

*x *is a real column vector of size *L *- 1

**initialization ***x *= (1,...,1)'

**main loop **for *i *= 1...*n *- 1 do

    • *x *= *R *× *x *(sparse product)

**end **return 

The more critical stage of both these algorithms is the sparse product of the matrix *R *by a column vector which can be efficiently done with *ζ *operations.

It is interesting to point out the fact that these algorithms do not require the stationarity of the underlying Markov chain. More surprisingly, it is also possible to relax the random sequence homogeneity assumption. Indeed, if our transition matrix ∏ depends on the position *i *in the sequence, we simply have to replace *R *in the algorithms with the corresponding *R*_*i *_(which may use a significant amount of additional memory depending on its expression as a function of *i*).

For complementary p-value, we require to compute *R*_1_*R*_2_...*R*_*n*-1_*R*_*n*_*x *which is easily done recursively starting from the right. In the direct p-value case however, it seems more difficult since we need to compute *x *+ *R*_1_*x *+ *R*_1_*R*_2_*x *+ ... + *R*_1_*R*_2_...*R*_*n*-1_*R*_*n*_*x*. Fortunately this sum can be rewritten as *x *+ *R*_1_(*x *+ *R*_2_{... [*x *+ *R*_*n*-1_(*x *+ *R*_*n*_*x*)]...}) which is again easy to compute recursively starting from the right.

The resulting complexities in the heterogeneous case are hence the same than in the homogeneous one (assuming that the number of non zero terms in *R*_*i *_remains approximately constant). This remarkable property of the FMCI should be remembered especially in the biological field where most sequences are known to have complex heterogeneous structures which are often difficult to take into account.

## 3 Application 1: local score

We propose in this part to apply our results to the computation of exact p-values for local score. We first recall the definition of the local score of one sequence (section 3.1) and design a FMCI allowing to compute p-value in the particular case of an integer and i.i.d. score (section 3.2). We explain in sections 3.5 and 3.6 how to relax these two restrictive assumptions to consider rational or Markovian scores. The main result of this part is given in section 3.4 where we propose an algorithm improving the simple application of the general ones by using a specific asymptotic behaviour presented in section 3.3. As numerical application, we propose finally in section 3.7 to find significant hydrophobic segments in the Swissprot database using the Kyte-Doolittle hydrophobic scale. Our exact results are compared to the classical Gumble asymptotic approximations and discussed both in terms of numerical performance and reliability.

### 3.1 Definition

We consider *S *= *S*_1_,...,*S*_*n *_a sequence of real scores and we define the local score *H*_*n *_of this sequence by



which is exactly the highest partial sum score of a subsequence of *S*.

This local score can be computed in *O*(*n*) using the auxiliary process

*U*_0 _= 0     and for 1 ≤ *j *≤ *n *     = max{0, *U*_*j*-1 _+ *S*_*j*_}     (10)

because we then have *H*_*n *_= max_*j *_*U*_*j*_.

Assuming the sequence *S *is random (Bernoulli or Markov model), we want to compute p-values relative to the event *E*_*n *_= {*H*_*n *_≥ *a*} where *a *> 0.

### 3.2 Integer score

In order to simplify, we will first consider the case of integer scores (and hence *a *∈ ≁) then we will extend the result to the case of rational scores.

In the Bernoulli case, [12] introduced the FMCI *Z *defined by



(resulting with a sequence of length *n *+ 1) with 0 as the only starting state and *a *as the final absorbing state. The transition matrix ∏ is given by



where

*p*(*i*) = ℙ(*S*_1 _= *i*)     *f*(*i*) = ℙ(*S*_1 _≤ *i*)     *g*(*i*) = ℙ(*S*_1 _≥ *i*)     ∀*i *∈ ℤ     (13)

It is possible to apply to this case the general algorithm 1 with *L *= *a *+ 1 and *k *= 1 (please note that we have added *Z*_0 _to the sequence and *n *must then be replaced by *n + *1 in the algorithm to get correct computations) to compute the p-value we are looking for. In the worst case, *R *has *ζ *= *a*^2 ^non zero terms and the resulting complexity is *O*(*a*^2^) in memory and *O*(*n *× *a*^2^) in times. But in most cases, *S*_1 _support is reduced to a small number of values and the complexities decrease accordingly.

### 3.3 Asymptotic development

Is it possible to compute this p-value faster ? In the case where *R *admits a diagonal form, simple linear algebra could help to cut off the computations and answer yes to this question.

**Proposition 4**. If *R *admits a diagonal form we have



where []_1 _denotes the first component of a vector, with *R*^∞ ^= lim_*i*→∞ _*R*^*i*^/*λ*^*i*^, where 0 <*λ *< 1 is the largest eigenvalue of *R *and *ν *is the magnitude of the second largest eigenvalue. We also have *v *= [*g*(*a*),...,*g*(1)]'.

*Proof*. By using the corollary 15 (appendix A) we know that

*R*^*i *^- *λ*^*i*^*R*^∞ ^= *O*(*ν*^*i*^)     (15)

uniformly in *i *so we finally get for all *α*



uniformly for all *n *≥ *α *and the proposition is then proved by considering the first component of equation (16).     □

**Corollary 5**. We have



and



*Proof*. Simply replace the terms in (17) and (18) with equation (14) to get the results.     □

### 3.4 Algorithm

The simplest way to compute ℙ(*H*_*n *_≥ *a*) is to use the algorithm 2 in our particular case. As the number of non zero terms in *R *is then *a*^2^, the resulting complexity is *O*(*n *× *a*^2^). Using the proposition 4, it possible to get the same result a bit faster on very long sequence by computing the first two largest eigenvalues magnitudes *λ *and *ν *(complexity in *O*(*a*^2^) with Arnoldi algorithms) and to use them to compute a p-value.

As the absolute error is in *O*(*ν*^*α*^) we obtain a require *ε *error level using a *α *proportional to log(*ε*)/log(*ν*) which results in a final complexity in *O*(log(*ε*)/log(*ν*) × *a*^2^). Unfortunately, this last method requires to use delicate linear algebra techniques and is therefore more difficult to implement. Another better possibility is to use the corollary 5 to get the following fast and easy to implement algorithm:

algorithm 3: local score p-value

*x *a real column vector of size *a*, (*p*_*i*_)_*i*≥1 _and (*λ*_*i*_)_*i*≥3 _to sequences of real and *i *an integer

**initialization ***x *= [*g*(*a*),...,*g*(1)]', *p*_1 _= *g*(*a*), and *i *= 0

**main loop **while (*i *<*n *and (*λ*_*i*_) has not yet converged towards *λ*)

    • *i *= *i *+ 1

    • *x *= *R *× *x *(sparse product)

    • *p*_*i *_= *p*_*i*-1 _+ *x*_1_

    • *λ*_*i *_= (*p*_*i *_- *p*_*i*-1_)/(*p*_*i*-1 _- *p*_*i*-2_) (if defined)

**end **• *p *= *p*_*i*_

    • if (*i *<*n*) then *p *= *p *+ (*p*_*i *_- *p*_*i*-1_) 

    • return *p*

At any step *i *of the main loop we have *p*_*i *_= ℙ(*H*_*i *_≥ *a*) and the final value taken by *i *is the *α *of proposition 4. One should note that only the last three terms of (*p*_*i*_)_*i*≥1 _and (for a simple convergence testing) the last two terms of (*λ*_*i*_)_*i*≥3 _are required by the algorithm.

### 3.5 Rational scores

What if we consider now a rational score instead of an integer one ? If we denote by  ⊂ ℚ the support of *S*_1_, let us define *M *= min_*i*∈ℕ_{*i* ⊂ ℤ}. Changing the scale of the problem by the factor *M *allows us to get back to the integer case:

ℙ(*H*_*n *_≥ *a*) = ℙ(*M H*_*n *_≥ *M a*)     (19)

This scale factor will obviously increase the complexity of the problem, but as the support cardinal (denoted *η*) is not changed during the process, the resulting complexities are *O*(*M *× *a *× *η*) in memory and *O*(*M *× *n *× *a *× *η*) in time (*n *could vanish from the time complexity thanks to the faster algorithm presented above).

For example, if we consider the Kyte-Doolittle hydrophobicity score of the amino-acids (see [10] and table 1), it takes only *η *= 20 values and *M *= 10, the resulting complexity to compute ℙ(*H*_*n *_≥ *a*) is then *O*(200 × *n *× *a*). If we consider now the more refined Chothia score ([4]), the scale factor increases from *M *= 10 to *M *= 100 and the resulting complexities are multiplied by 10.

**Table 1:** Distribution of amino-acids estimated on Swissprot (release 47.8) database and Kyte-Doolittle hydrophobic scale. Mean score is -0.244.

a. a.	F	M	I	L	V	C	W	A	T	G
ℙ in %	4.0	2.4	5.9	9.6	6.7	1.5	1.2	7.9	5.4	6.9
score	2.8	1.9	4.5	3.8	4.2	2.5	-0.9	1.8	-0.7	-0.4
a. a.	S	P	Y	H	Q	N	E	K	D	R

ℙ in %	6.9	4.8	3.1	2.3	3.9	4.2	6.6	5.9	5.3	5.4
score	-0.8	-1.6	-1.3	-3.2	-3.5	-3.5	-3.5	-3.9	-3.5	-4.5

### 3.6 Markov case

All these results can be extended to the Markov case but this require to define a new FMCI allowing us to trace the last score (in the case of an order one Markov chain for the sequence *S*, if a higher order *m *is considered, we just have to add the corresponding number of preceding scores to *Z *instead of one):



Doing this now we get *k *= *η *(the cardinal of the score support) starting states instead of one so we need a starting distribution *μ *(which could be a Dirac) to compute the p-value.

We will not detail here the structure of the corresponding sparse transition matrix ∏ (see [13]) but we need to know its number *ζ *of non zero terms. If *a *is an integer value (we suppose here that the scale factor has been already included in it) then the order of *R *is *M *× *a *× *η *and *ζ *= *O*(*M *× *a *× *η*^2^) (and we get *O*(*M *× *a *× *η*^*m*+1^) when an order *m *Markov model is considered).

### 3.7 Numerical results

**Figure 1:**
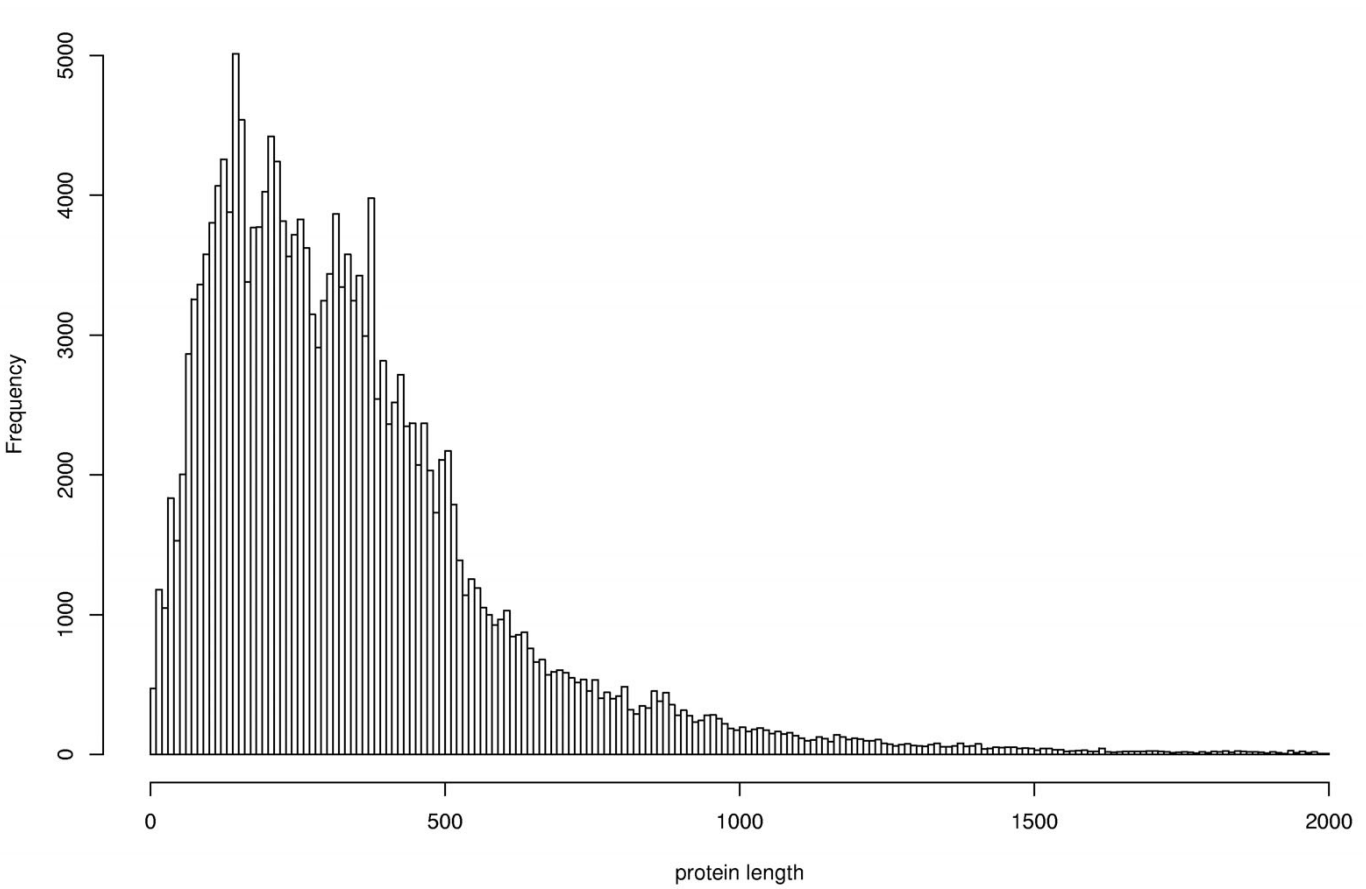
Empiric distribution of Swissprot (release 47.8) protein lengths. In order to improve readability, 0.5% of sequences with length ∈ [2 000, 9 000] have been removed from this histogram.

In this section, we apply the results presented above to a practical local score study. We consider the complete protein database of Swissprot release 47.8 and the classical amino acid hydrophobic scale of Kyte-Doolittle given in table 1 ([10]). The database contains roughly 200 000 sequences of various lengths (empiric distribution given in figure 1).

Once the best scoring segment has been determined for each of these sequences, we need to compute the corresponding p-values. According to [9], the asymptotic distribution of *H*_*n *_is given (if mean score is < 0, which is precisely the case here) by the following conservative approximation:

ℙ(*H*_*n *_≥ *a*) ≃ 1 - exp (-*nKe*^-*aλ*^)     (21)

where constants *λ *and *K *depend on the scoring distribution.

With our hydrophobic scale and a distribution of amino-acids estimated on the entire database we get

*λ *= 5.144775 × 10^-3 ^    and     *K *= 1.614858 × 10^-2^

(computation performed with a C function implemented by Altschul). Once the constants are computed we could get all the approximated p-values very quickly (a few seconds for the 200 000 p-values).

On the other hand, our new algorithm allows to compute (for the very first time) the exact p-values for this example. As the chosen scoring function has a one digit precision level, we need to use a scale factor of *M *= 10 to fall back to the integer case. A C++ implementation (available on request) performed all the computations in roughly three hours on a Pentium 4 CPU 2.8 GHz (this means approximately 20 p-values computed by second).

**Figure 2:**
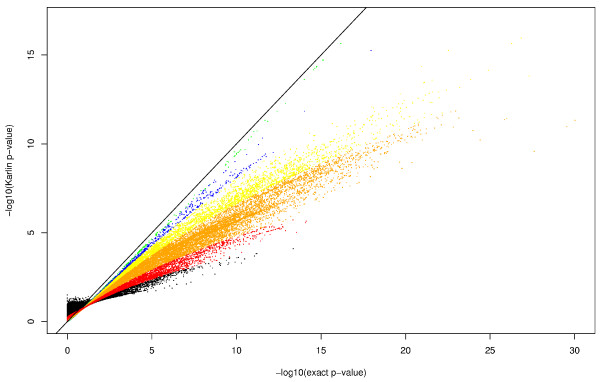
Exact p-value against Karlin ones (in log scale). Color refers to a range of sequence lengths: smaller than 100 in black (≃ 20 000 sequences), between 100 and 200 in red (≃ 40 000 sequences), between 200 and 500 in orange (≃ 90 000 sequences), between 500 and 1000 in yellow (≃ 30 000 sequences), between 1000 and 2 000 in blue (≃ 6 000 sequences) and greater than 2 000 in green (≃ 1 000 sequences). The solid line represents *y *= *x*. Range have been chosen for readability and few dots with exact p-value smaller than 10^-30 ^are hence missing.

We can see on figure 2 the comparison between exact values and Karlin's approximations. The conservative design of the approximations seems to be successful except for very short unsignificant sequences. While the approximations are rather close to perfection for sequences with more than 2 000 amino-acids, the smaller the sequence is, the worse the approximations get. This is obviously consistent with the asymptotic nature of Karlin's formula but seems to indicate that these approximations are not reliable for 99.5% of the sequence in the database (protein of length < 2 000).

One should object that it exists ([1,2]) a well known finite size correction to formula (21) that might be useful, especially when considering short sequences. Unfortunately in our case, this correction does not seems to improve the quality of the approximations (data not shown) and we hence make the choice to ignore it.

In table 2 we compare the number of sequences predicted to have a significant hydrophobic segment at a certain e-value level by the two approaches. If the Karlin's approximations are used, many proteins are considered unsignificant while they are. For example, with the classical database threshold of 10^-5^, only few sequences (6%) are correctly identified by Karlin's approximations.

**Table 2:** Number of e-value smaller than a threshold are given for exact computations (exact) and asymptotic Karlin's approximations (Karlin). The last row gives the accuracy of asymptotic predictions (accuracy = Karlin/exact).

e-value	10^-1^	10^-2^	10^-3^	10^-4^	10^-5^	10^-6^
exact	9473	7772	6271	4563	3232	2348
Karlin	3417	2047	1056	439	195	96
accuracy	34%	26%	17%	10%	6%	4%

We have seen that Karlin's approximations are often far too conservative to give accurate results, but what about the ranking ? Table 3 proposes the Kendall's tau rank correlation (see [16] chapter 14.6 for more details) which is equal to 1.0 for a complete rank agreement and equal to -1.0 for a complete inverse rank agreement. As we will certainly be interested in the most significant sequences produced by our study, we compute our Kendall's tau only on these sequences. When all sequence lengths are considered, Karlin's approximations show their total irrelevance to give correct ranking for the first 10 or 50 most significant p-values. Even when the 100 first p-values are taken into account, relative ranks given by Karlin's approximations are wrong in 63% of the cases, which is huge. However, in the case where the approximations values are close to the exact ones (sequence lengths greater than 2 000, which correspond only to 0.5% of the database), p-values obtained with both methods are highly correlated.

**Table 3:** Kendall's tau (rank correlation) comparing the most significant exact p-values (the reference) to the Karlin's approximations. The column "all" gives the result for all sequences while the R_*i *_give the results for a certain range of sequence lengths: smaller than 100 for R1, between 100 and 200 for R2, between 200 and 500 for R3, between 500 and 1 000 for R4, between 1 000 and 2 000 for R5 and greater than 2 000 for R6.

number of p-values	all	R1	R2	R3	R4	R5	R6
10	0.30	0.64	0.24	-0.20	0.58	0.64	0.97
50	0.14	0.73	0.50	0.46	0.56	0.78	0.97
100	0.37	0.70	0.67	0.62	0.61	0.80	0.98

## 4 Application 2: pattern statistics

In this part, we consider the application of FMCI to pattern statistics. After a short introduction of notations (section 4.1) we explain with an example in section 4.2 how to build through the tool of DFA a particular FMCI related to a given pattern. The block structure of this FMCI (section 4.3) is then used to get in section 4.4 two efficient algorithms for under- and over-represented patterns. We derive in section 4.5 some asymptotic developments but unlike with local score application, these results are not used to improve our algorithms. In the last section 4.6 we finally compare this new method to existing ones.

### 4.1 Definition

Let us consider a random order *m *homogeneous Markov sequence *X *= *X*_1_,...,*X*_*n *_on the finite alphabet  (cardinal *k*). If *N*_*i *_is the random variable counting the number of occurrences (overlapping or renewal) of a given pattern in *X*_1_...*X*_*i*_. We define the pattern statistic associated to any number *N*_obs _∈ ℕ of observations by



This way, a pattern has a positive statistic if it is seen more than expected, a negative statistic if seen less than expected and, in both cases, the corresponding p-value is given (in log scale) by the magnitude of the statistic.

The problem is: how to compute this statistic ?

### 4.2 DFA

We first need to construct a Deterministic Finite state Automaton (DFA) able to count our pattern occurrences. It is a finite oriented graph such as all vertexes have exactly *k *arcs starting from them each one tagged with a different letter of . One or more arcs are marked as counting ones. By processing a sequence *X *in the DFA, we get a sequence *Y *(of vertexes) in which the words of length 2 corresponding to the counting transitions occur each time a pattern occurs in *X*.

Example: If we consider the pattern aba.a (. means "any letter") on the binary alphabet  = {a, b}. We define vertex set  = {a, b, ab, aba, abaa, abab} and then the structure of the DFA counting the overlapping occurrences (set of vertexes and structure would have been slightly different in the renewal case) of the pattern is given by



(the counting arcs are denoted by a star). In the sequence



of length *n *= 20, the pattern occurrences end in positions 9,11 and 18. Processing this sequence into the DFA gives



which is a sequence of the same length as *X*, where occurrences of the pattern end exactly in the same positions.

If *X *is an homogeneous order one Markov chain, so is *Y *and its transition matrix is given by *P *+ *Q *where *P *contains the non counting transitions and *Q *the counting ones:



and



It is therefore possible to work on *Y *rather than on *X *to compute the pattern statistics. In order to do that, it is very natural to use the large deviations (in this case, computations are closely related to the largest eigenvalue of the matrix *T*_*θ *_= *P *+ *Qe*^*θ*^) but other methods can be used as well (binomial or compound Poisson approximations for example).

This method easily extends to cases where *X *is an order *m *> 1 Markov chain by modifying accordingly our vertex set. For example, if we consider an order *m *= 2 Markov model our vertex set becomes

 = {aa, ab, ba, bb, aba, abaa, abab}

In all cases, if we denote by *L *the cardinal of . In order to count overlapping occurrences of a non degenerate pattern of length *h *on a size *k *alphabet we get *L *= *k *+ *h *- 2 when an order 1 Markov model is considered and *L *= *k*^*m *^+ *h *- *m *- 1 for an order *m *> 1 Markov model. For a degenerate pattern of length *h*, *L *is more difficult to know as it depends on the degeneracy of the patterns, in the worst case *L *= *k*^*h*-1^, but *L *should be far smaller in most cases. One should note that *L *increases by the number of different words present in the pattern if we consider renewal occurrences instead of overlapping ones.

Although construction and properties of DFA are well known in the theory of language and automata ([8]), their connexions to pattern statistics have surprisingly not been extensively studied in the literature. In particular, the strong relation presented here between the FMCI technique for pattern and DFA appears to have never been highlighted before. If this interesting subject obviously need to (and will soon) be investigated more deeply, it is not really the purpose of this article which focus more on the algorithmic treatment of a built FMCI.

### 4.3 FMCI

Once a DFA and the corresponding matrices *P *and *Q *have been built, it is easy to get a FMCI allowing to compute the p-values we are looking for.

Let us consider



where *Y*_*j *_is the sequence of vertexes, *N*_*j *_is the number of pattern occurrences in the sequence *Y*_1_...*Y*_*j *_(or *X *= *X*_1_...*X*_*j *_as it is the same), where *f *is the final (absorbing state) and where *a *∈ ℕ is the observed number of occurrences *N*_obs _if the pattern is over-represented and *N*_obs _+ 1 if it is under-represented.

The transition matrix of the Markov chain *Z *is then given by:



where for all size *L *blocks *i*, *j *we have



with Σ_*Q*_, the column vector resulting from the sum of *Q*.

By plugin the structure of *R *and *v *in the corollaries 2 and 3 we get the following recurrences:

**Proposition 6**. For all *n *≥ 1 and 1 ≤ *i *≤ *k *we have



where for *x *= *u *or *v *we have ∀*j *≥ 0 the following size *L *block decomposition:  and we have the recurrence relations:



with *u*^0 ^= (1...1)' and *v*^0 ^= *v*.

### 4.4 Algorithms

Using the proposition 6 it is possible to get an algorithm computing our pattern statistic for an under-represented pattern observed *N*_obs _times:

algorithm 4under: exact statistics for under-represented pattern

*x*_0_,..., and *y*_0_,..., are 2 × (*N*_obs _+ 1) real column vectors of size *L*

**initialization **for *j *= 0...*N*_obs _do *x*_*j *_= (1,...,1)'

**main loop **for *i *= 1...(*n *- 1) do

    • for *j *= 0...*N*_obs _do *y*_*j *_= *x*_*j*_

    • *x*_0 _= *P *× *y*_0_

    • for *j *= 1...*N*_obs _do *x*_*j *_= *P *× *y*_*j *_+ *Q *× *y*_*j*-1_

**end **• 

    • return log_10_(*q*)

If we consider now an over-represented pattern we get

algorithm 4over: exact statistics for over-represented pattern

*x*_1_,...,, *y*_1_,..., and *z *are 2*N*_obs _+ 1 real column vectors of size *L*

**initialization ***z *= (0,...,0)', *x*_1 _= Σ_*Q *_and for *j *= 2...*N*_obs _do *x*_*j *_= (0,...,0)'

**main loop **for *i *= 1...(*n *- 2) do

    • for *j *= 1...*N*_obs _do *y*_*j *_= *x*_*j*_

    • *x*_1 _= *P *× *y*_1_

    • for *j *= 2...*N*_obs _do *x*_*j *_= *P *× *y*_*j *_+ *Q *× *y*_*j*-1_

    • *z *= *z *+ 

**end **• 

    • return -log_10_(*p*)

As we have *O*(*k *× *L*) non zero terms in *P *+ *Q*, the complexity of both of these algorithms is *O*(*k *× *L *+ *N*_obs _× *L*) in memory and *O*(*k *× *L *× *n *× *N*_obs_) in time.

To compute p-values out of floating point range (ex: smaller than 10^-300 ^with C double), it is necessary to use log computations in the algorithms (not detailed here). The resulting complexity stays the same but the empirical running time is obviously slower. That is why we advise to use log-computation only when it is necessary (for example by considering first a rough approximation).

### 4.5 Asymptotic developments

In this part we propose to derive asymptotic developments for pattern p-values from their recursive expressions. For under- (resp. over-) represented patterns, the main result is given in theorem 9 (resp. 12). In both cases, theses results are also presented in a simpler form (where only main terms are taken into account) in the following corollaries.

**Proposition **7. For any *x *= (*x*_(*a*-1)_,...,*x*_0_)' and all *β *≥ 0 *x*^*β *^= *R*^*β*^*x *is given by  = *P*^*β*^ and



*Proof*. As  =  for all *j *≤ 0 it is trivial to get the expression of . If we suppose now that the relation (28) is true for some *i *and *β *then, thanks to the relation (27) we have



and so the proposition is proved through the principle of recurrence.     □

**Lemma 8**. For all *i *≥ 0 and *a *≤ *b *∈ ℕ and *r *> 0 we define



If *r *≠ 1 we have for all *i *≥ 0 we have



and (case *r *= 1) for all *i *≥ 0 we have



*Proof*. Easily derived from the following relation



**Theorem 9**. If *P *is primitive and admits a diagonal form we denote by *λ *> *ν *the largest two eigenvalues magnitude of *P *by *P*^∞ ^= lim_*i*→+∞ _*P*^*i*^/*λ*^*i *^(a positive matrix) and we get for all *α *≥ 1 and *i *≥ 0



uniformly in *β *and where  is a polynomial of degree *i *which is defined by  and for all *i *≥ 1 by the following recurrence relation:



*Proof*. See appendix B.     □

**Corollary 10**. With the same assumptions than in the theorem 9, for all *α *≥ 1 and *β *≥ (*i*+1)*α *we have



*Proof*. Equation (37) and the lemma 8 gives



and the result is then proved by a simple recurrence.     □

**Proposition 11**. For any *x *= (*x*_(*a*-1)_,...,*x*_0_)' and all *β *≥ 0,  is given by  and



*Proof*. Using equation (28) we get



which gives the proposition.     □

From this result (very similar to proposition 7) it is possible to get a new theorem

**Theorem 12**. If *P *is primitive and admits a diagonal form we denote by *λ *> *ν *the largest two eigenvalues magnitude of *P *by *P*^∞ ^= lim_*i*→+∞ _*P*^*i*^/*λ*^*i *^(a positive matrix) and we get for all *α *≥ 1 and *i *≥ 0



uniformly in *β *and where  is a constant term defined by



and for all *i *≥ 0 by the following recurrence relation



and  is a polynomial of degree *i *which is defined by



and for all *i *≥ 1 by the following recurrence relation:



*Proof*. Easy to derive from the proof of theorem 9.     □

**Corollary 13**. We have the same assumptions than in the the theorem 12, for all *α *≥ 1 and *β *≥ (*i *+ 1)*α *we have



*Proof*. Easy to derive from the proof of corollary 10.     □

### 4.6 Numerical results

We propose here to consider numerical applications of these new FMCI pattern statistics algorithms and to compare their results and performance to exact computations using Simple Recurrences ([17] and [18]) denoted SR from now.

All computations are performed using SPatt-1.2.0 package (see [14] or [15]) on a 2.7 Gz P4 with 512 Mo running the linux 2.6.8 system.

As we can see on table 4, the computational time to obtain the pattern statistics for all simple words of a given length are quite similar with both approaches. One exception: the Bernoulli case (*m *= 0) where SR are roughly 10 times faster than FMCI computations. This is due to the fact that the Bernoulli case uses simpler recurrences than the ones used for the true Markovian cases (*m *≥ 1). Similar simplifications in the DFA structures can reduce the computational time of FMCI approach in the independent case but they have not been implemented here (as their use is often marginal).

**Table 4:** Computational time (in seconds) to get the statistics of all DNA words of length *h *in the HIV complete genome sequence (*n *= 9719) using either simple recurrences of finite Markov chain imbedding and in respect with an order *m *Markov model estimated on the genome.

Markov order word length	*m *= 0			*m *= 1			*m *= 2		
	*h *= 3	*h *= 4	*h *= 5	*h *= 3	*h *= 4	*h *= 5	*h *= 3	*h *= 4	*h *= 5
SR	3	4	5	61	59	62	104	102	106
FMCI	39	45	52	39	44	52	96	102	113

If we consider now degenerate patterns instead of simple words (see table 5), FMCI approach clearly outperforms the SR one. Nevertheless, as considering degenerated patterns roughly multiply their observed number of occurrences by the alphabet size for each inde-termination, the corresponding computational time grows in the same manner which usually limits the use of high degenerated patterns in practical cases.

**Table 5:** Computational time (in seconds) to get the statistics of degenerate patterns (the dot means "any letter") occurring 100 times in an order *m *= 1 Markovian sequence of length *n *= 9719 which parameters are estimated on the HIV complete genome sequence using either simple recurrences of finite Markov chain imbedding.

pattern	atgca	at.ca	at..a	a...a
SR	0.60	8.20	2438.43	105 209.12
FMCI	0.51	1.15	3.01	91.80

Another interesting point is the memory requirements of the two approaches. Exact computations using SR have a *O*(*n *+ *α *× *k*^2*m*^) memory complexity where *n *is the sequence length, *k *the alphabet size, *m *the Markov model order and *α *which depends on the convergence rate of the model towards its stationary distribution. As a consequence, SR is difficult to use in practice with *m *> 3 for DNA words or *m *> 1 for protein ones. For FMCI computations, the memory requirements remain very cheap and in practice, any Markov model that fit in memory can be considered.

What about the reliability of the two methods. Once the pattern DFA has been computed, the FMCI algorithms are very simple to implement and have a high numerical stability. On the other hand, SR algorithms are quite more complicated (especially for degenerated patterns) to implement and require to approximate the iterate power of the Markov transition by the stationary distribution for large iterates. Classical convergence issues could result then to some numerical instability when high Markov orders are considered. As a consequence, FMCI results are taken as references from this point.

In table 6 we can see that for p-values larger than 10^-300 ^the results given by both methods are exactly the same when we consider order 0 Markov models. As smaller p-values are not well managed by C double precision computation (the exact limit depends on the system), we get wrong results unless log computations are used. Such computations have been implemented for FMCI algorithms (they are quite simple) but not for SR ones (where it is quite more complicated) which explain the differences for patterns at and tcgatc.

**Table 6:** Reliability of pattern statistics. They are computed in respect with an order *m *Markovian sequence of length *n *= 9719 which parameters are estimated on the HIV complete genome. Relative error uses FMCI statistics as reference.

pattern	order	observed	expected	FMCI	SR	relative error
acta	0	106	48.63	+12.208	+12.208	0.0
acta	1	106	47.01	+13.090	+13.079	8.7 × 10^-4^
acta	0	26	48.63	-3.567	-3.567	0.0
acta	1	26	47.01	-3.231	-3.230	4.8 × 10^-4^
acta	0	6	48.63	-13.856	-13.850	3.7 × 10^-4^
acta	1	6	47.01	-13.237	-13.237	3.0 × 10^-5^
at	0	50	759.48	-291.610	-291.610	0.0
at	0	25	759.48	-327.214	-318.192	2.8 × 10^-2^
at	0	0	759.48	-377.009	-319.607	1.5 × 10^-1^
tcgatc	0	185	1.37	+294.997	+294.997	0.0
tcgatc	0	195	1.37	+314.388	+314.388	5.7 × 10^-8^
tcgatc	0	205	1.37	+333.931	na	na
acacaa	2	10	6.66	+0.865	+0.855	1.1 × 10^-2^
acacaa	2	20	6.66	+4.669	+4.520	3.2 × 10^-2^
acacaa	2	60	6.66	+35.751	+33.532	6.2 × 10^-2^
acacaa	2	100	6.66	+79.736	+73.451	7.8 × 10^-2^

**Table 7:** 

tag\vertex	a	b	ab	aba	abaa	abab
a	a	a	aba	abaa	a*	aba*
b	ab	b	b	abab	ab	b

When we consider order *m *> 0 Markov models, the numerical approximations done on the iterate power of the transition matrix lead to some errors. For order 1 Markov model, these errors remain quite small, but when order 2 model are considered it is more sensitive. In both cases, the larger the statistic to compute is, the greater the errors made are.

## 5 Conclusion

We proposed in this paper two general algorithms allowing to compute quickly and in a stable numerical way any p-value that can be imbedded in a finite Markov chain. We used these algorithms in two applications: local score on one sequence and pattern statistics.

For local score, the resulting algorithms reduce dramatically the complexity of previously proposed naive ones allowing for the very first time to produce exact computations for practical biological studies (Kyte-Doolittle hydrophobic scale on the Swissprot database). Comparing the results to the classical and very popular Karlin's approximations, it appears that these approximations require long sequences (length greater than 2 000) which can dramatically reduce their range of applicability (only 0.5% of the data in our example). Of course, the exact computations require more time than the approximations, but are nevertheless fast enough (20 p-value per second in our example) to be used in most practicable cases. As a consequence we strongly advise to replace asymptotic approximations by these new exact ones whenever it is possible.

Concerning pattern statistics, the new FMCI algorithms appear to outperform the existing ones ([17]) in all possible ways: far easier to implement, more numerical stability, less memory requirements, as fast as SR for simple words (except in the M0 case, but this is due to a poor implementation of this particular case in FMCI approach) and dramatically faster (up to 1 000 times and more) for degenerated patterns. Even if the SR algorithms remain available in the SPatt package, FMCI ones are now used by default for exact computations.

## Appendix A: power of a sub-stochastic matrix

**Proposition 14**. If *P *is an order *L *irreducible sub-stochastic matrix admitting a row-eigenvector basis (*e*_1_,...,*e*_*L*_) where each *e*_*j*_, is associated to the eigenvalue *λ*_*j *_and |*λ*_1_| ≥ |*λ*_2_| ≥...≥ |*λ*_*L*_| then we have



where *λ *= |*λ*_1_| = *λ*_1_, *P*^∞ ^=  and ∀*j*



*Proof*. For any vector  we have



As *P *is an irreducible, Perron-Frobénius theorem (see [3] for example) assure that *λ *is real and the sub-stochastic property, that *λ *≤ 1 (in the particular case where *P *is also primitive *i.e. *∃*m*, *P*^*m *^> 0 then |*λ*_2_| <*λ*) Replacing *x *by *I*_ℓ _equation (51) gives the expression of the row ℓ of *P*^*i *^and the proposition is then proved.     □

**Corollary 15**. If *P *is an order ≥ 2 irreducible sub-stochastic matrix admitting a diagonal form then there exists a matrix *P*^∞ ^such as

*P*^*i *^- *λ*^*i*^*P*^∞ ^= *O*(*ν*^*i*^)     (52)

uniformly in *i *and where 1 ≥ *λ *≥ *ν *are the two largest eigenvalues magnitudes. In the special case where *P *is primitive, then *λ *> *ν *and *P*^∞ ^= lim_*i*→+∞ _*P*^*i*^/*λ*^*i *^is a positive matrix.

*Proof*. Using proposition 14 we get



and the corollary is proved.     □

## Appendix B: proof of theorem 9

Using propositions 7 and 14 we get the result for *i *= 0. Let us prove the theorem by the principle of recurrence. We assume now that the result is true until rank *i *- 1. Computing  for all *β *≥ (*i *+ 1)*α *we get



For all 1 ≤ *j *≤ *iα *we have *β *- *j *≥ *β *- *iα *≥ *α *so we get



For all *iα *+ 1 ≤ *j *≤ *β *- *α *we have *j *- 1 ≥ *iα *and *β *- *j *≥ *β *- *iα *≥ *α *and so, with the help of lemma 8 we get



thanks to the recurrence assumption, it is easy to see than *B *contribute with polynomial terms of degree *i *(as we have a sum on *O*(*β*) terms).

For all *β *- *α *+ 1 ≤ *j *≤ *β *we have *j *- 1 ≥ *iα *so we get



*C *contributing with polynomial terms of degree *i *- 1 (as we have a sum on *α *terms) Summing up all terms we get the result at rank *i *and the theorem is proved.
